# The role and reach of alcohol reduction apps

**DOI:** 10.1093/haschl/qxaf034

**Published:** 2025-02-14

**Authors:** Lori Uscher-Pines, Jessica L Sousa, Ateev Mehrotra, Alisa B Busch, Constance M Horgan, Haiden A Huskamp

**Affiliations:** RAND Corporation, Arlington, VA 22202, United States; RAND Corporation, Boston, MA 02116, United States; Department of Health Services, Policy and Practice, Brown University School of Public Health, Providence, RI 02912, United States; Department of Health Care Policy, Harvard Medical School, Boston, MA 02115, United States; Health Services Research Division, McLean Hospital, Belmont, MA 02478, United States; Institute for Behavioral Health, Heller School for Social Policy and Management, Brandeis University, Waltham, MA 02453, United States; Department of Health Care Policy, Harvard Medical School, Boston, MA 02115, United States

**Keywords:** alcohol misuse, health apps, alcohol reduction apps, alcohol use disorder

## Abstract

Although apps are widely available and have several advantages as a tool to support alcohol reduction and recovery, little is known about how individuals are using them. In 2024, we conducted an exploratory sequential mixed-methods study that coupled in-depth interviews with 22 app users and a nationally representative survey of 2002 adults. We explored experiences with and perceptions of alcohol reduction apps. Approximately 6% of US adults in the sample reported using alcohol reduction apps, and for most, it was the only support to address problematic drinking. In interviews, some users viewed apps as an alternative to traditional services and a way to independently address alcohol use; however, apps were seen as disconnected from care even by those who also used other supports. App users accessed a variety of features, with the most common being motivational content, tracking alcohol consumption, and educational content. Findings suggest that there are opportunities to not only introduce apps to individuals receiving healthcare services or participating in mutual support groups, but to reference and embed them in those settings. New approaches to regulation and reimbursement may support adoption as well as integration into healthcare services.

## Introduction

Alcohol is a major public health problem, with 1 in 4 US adults reporting binge drinking in the past 30 days^1^ and 11% meeting criteria for alcohol use disorder (AUD).^2^ Further, alcohol misuse is under-treated for a number of reasons including but not limited to stigma, lack of awareness among clinicians of how to identify problematic use and treatment options, and co-occurring mental health issues that can complicate diagnosis and treatment.^3-5^ Health apps could be 1 mechanism to increase treatment. Broadly defined as software applications that perform a specific health-related task or function on a mobile device, health apps of some type are now used by the majority of Americans^6^ and have the potential to support behavior change on a large scale.^7^ There are hundreds of alcohol reduction apps that help individuals reduce their alcohol intake, abstain from alcohol, and/or support long term recovery available in app stores.^8,9^

Alcohol reduction apps typically work in 2 key ways: (1) self-monitoring of user behavior and/or (2) providing social support.^10^ While there is limited assessment of their effectiveness,^9,11^ alcohol reduction apps have many potential advantages. In contrast to traditional healthcare services, apps can provide support in real time. They can also reach individuals who are not engaged in care or those in precontemplation or contemplation stages of behavior change. Further, apps are inexpensive, widely available, and offer anonymity to those who would be deterred from accessing services because of stigma.^10,12^

Despite the promise of apps as a scalable tool for reducing alcohol-related harms, little research has explored how often and in what ways individuals are using alcohol reduction apps. To explore and quantify individuals’ experiences with these apps, we applied an exploratory sequential mixed-methods study design that included qualitative interviews with app users and a nationally representative survey.^13^

## Methods

### Overview

In exploratory sequential designs, qualitative results are used to develop a new instrument or taxonomy for the quantitative component.^13^ In this study, we first conducted interviews with individuals who had used 1 or more alcohol reduction apps in the prior 2 years. Then, we fielded a cross-sectional survey to a national panel of US adults. The goal of interviews was to explore experiences with and perceptions of alcohol reduction apps in depth and to inform the design of the survey, whereas the goal of the survey was to generate nationally representative data on the prevalence of app use. In this study, alcohol reduction apps represent a broad category of apps that include those focused on alcohol reduction and/or cessation as well as those supporting recovery. We analyzed quantitative and qualitative data separately and used a contiguous approach to juxtapose and integrate the data through narrative.^14^ This study was approved by RAND's Institutional Review Board.

### In-depth interviews

To support interview recruitment, we engaged a healthcare market research firm with an online research panel of over 1 million US adults. The study description and eligibility screener were shared with a random sample of approximately 31 000 panelists. Of the 5999 individuals who responded to the eligibility survey, 108 met our inclusion criteria. To be eligible, a participant had to (1) report using 1 or more mobile phone app(s) in an effort to reduce their drinking or support them in their sobriety and (2) be 18-85 years of age. Among those eligible, we conducted heterogeneity sampling, recruiting 22 app users who varied with respect to gender, age, race and ethnicity, and US region.

Participants participated in 60-min semi-structured interviews and received a $85 Amazon gift card. The interview protocol covered the number and types of apps used, usage patterns (eg, frequency and timing of use), features, user satisfaction, app strengths and weaknesses, costs to download and use apps, and the role of apps in overall care. Participants provided oral informed consent. Two team members trained in qualitative research (L.U.-P., J.L.S.) conducted the interviews, which were recorded via Zoom and manually transcribed.

We conducted a rapid thematic analysis using matrices and deductive coding.^15^ Interview notes were entered into a spreadsheet after each interview, with quotes organized for each participant (row) by interview question (columns). L.U.-P. and J.L.S. reviewed the matrix and presented preliminary themes for discussion and refinement by the larger research team.

### National survey

We fielded the survey to the American Life Panel (ALP), a probability-based sample of US adults who are provided with internet-connected devices and incentives to complete surveys on a recurring basis (see details reported elsewhere).^16^ Our research team added dedicated questions to the ALP Omnibus Survey fielded in October 2024. The selection and wording of questions and response options were informed by interview findings. Of the 2876 ALP panelists invited to participate, 2002 completed the survey, for a response rate of 69.6%.

We defined mobile apps for survey participants as follows: *A mobile application or app is a computer program or software application designed to run on a smartphone or tablet. Some people use mobile phone apps to help with behavior change. For example, people might use apps to help lose weight, quit smoking, or drink less alcohol.* We first asked about the number of apps used (if any) in the past 2 years to help reduce drinking or support sobriety. Among those who reported app use, we asked what features of the app(s) they had accessed. Further, app users were asked whether app(s) were the only tool they had used or they had used app(s) along with other tools, services, and/or supports for alcohol use (eg, therapy, Alcoholics Anonymous) (Table 1).

**Table 1.** Survey questions.

A mobile application or “app” is a computer program or software application designed to run on a smartphone or tablet. Some people use mobile phone apps to help with behavior change. For example, people might use apps to help lose weight, quit smoking, or drink less alcohol.
In the past 2 years, have you used an app specifically to help with drinking or sobriety (for example, to track alcohol use)? Select one response. □ Yes, I have used 1 app. □ Yes, I have used 2 or more apps. □ No, I have not used any apps for drinking or sobriety.
What features of the drinking or sobriety app(s) did you use in the past 2 years? Select all that apply. □ Connection to peers (support, encouragement, or advice) □ Educational content (articles, videos) □ Motivational content (quotes, anecdotes) □ Resources (therapist directory, meeting lists) □ Tracking number of drinks consumed □ Tracking sobriety milestones (eg, days sober) □ Online therapy or meetings with healthcare providers □ Other (please specify): ____________
Which of the following best describes the role app(s) have played in your drinking or sobriety services in the past 2 years? Select one response. □ App(s) are the only tool or support I have used. □ I have used apps along with other tools, services, and/or supports (for example, therapy, Alcoholics Anonymous).

This table depicts the survey questions that were developed by the research team and fielded through the RAND American Life Panel Omnibus Survey in October 2024.

We calculated descriptive statistics using sampling weights to produce nationally representative estimates. Sampling weights matched sample demographics to the US population and accounted for nonresponse. Missing data were limited (<1% of all variables) and likely random; participants with missing data for a specific survey question were excluded from analyses of that question, but were included in analyses for other questions they answered. Analyses were conducted using Stata, version 17.

### Limitations

This study had several limitations. First, interview participants opted to participate in an online panel; it follows that they could have higher digital literacy than the general population of individuals with problematic drinking and may use apps differently than individuals facing more technology-related challenges. Second, given the relatively low rate of app use in the ALP sample, we were limited in the types of analyses we could conduct (eg, subgroup analyses). Third, we did not obtain data about participants’ current or prior alcohol use, and only the interviews (not the survey) discussed individuals’ goals with apps (eg, to support abstinence, reduce number of drinks consumed per week). It is possible that some survey respondents used alcohol reduction apps as part of broader lifestyle interventions (eg, to be more healthy, lose weight) and not for alcohol misuse. Further, we did not have a large enough sample size in interviews to explore whether themes differed for individuals who had different goals with app use.

## Results

### Interview and survey sample characteristics

Of the 22 interview participants, 13 (59.1%) were female and 9 (40.1) identified as Black, Hispanic, Asian, Native American, and/or multi-racial. Nine (40.1%) had Medicaid insurance, and the mean age was 38.5 years. Interview participants (*N* = 22) represented all US regions (data not shown).

Survey respondents (*N* = 2002) had a mean age of 49.0 years, and 19.6% self-identified as Hispanic, 12.9% as Black, and 75.6% as White. Among respondents, 48.8% were female (Table 2).

**Table 2.** Characteristics of American Life Panel survey participants and app users, 2024.

	All participants (*N* = 2002)		Participants who used app(s) for drinking (*n* = 66)	
Characteristics	Frequency (unweighted)	Percent (weighted)	Frequency (unweighted)	Percent (weighted)
Sex				
Female	1120	48.8	32	42.9
Male	882	51.2	34	57.1
Age group, years				
20-39	184	34.8	21	61.7
40-59	624	33.3	33	33.4
≥60	1194	31.8	12	4.9
Race^a^				
Black	174	12.9	10	16.6
White	1639	75.6	45	75.1
Other^b^	189	11.5	11	8.3
Ethnicity^a^				
Latino	257	19.6	14	24.0
Household income				
<$35 000	387	19.1	20	29.7
$35 000-$99 999	901	44.3	22	34.2
≥$100 000	714	36.6	24	36.1
Urbanicity				
Rural or small town (population <50 000)	483	24.1	11	21.3
Small to midsize or large city (population ≥ 50 000)	1518	75.9	55	78.7

The table shows the demographic characteristics of all survey participants and of the subset who used apps for problematic drinking in the prior 2 years. Source: Authors’ analysis of RAND American Life Panel survey data, October 2023.

^a^Race and ethnicity were self-reported by survey participants, who chose from a set of race and ethnicity options defined by American Life Panel investigators.

^b^Includes Asian or Pacific Islander, American Indian or Alaskan Native, or other race.

### Interview findings

Interview participants together mentioned experiences with over 30 apps to reduce drinking and/or support recovery. The apps most frequently used by participants included Reframe (*n* = 8 participants), I am Sober (*n* = 5), and Sunnyside (*n* = 5). Most participants had used more than 1 app, and several described an initial trial period where they downloaded multiple apps on their smartphone to explore which ones might work best for them. About half of participants reported that they were currently using apps “often” or “always.”

Multiple participants also described using apps more frequently in the early stages of sobriety or behavioral change, with waning use over time. As 1 participant from North Carolina explained, “I used them [apps] predominantly in the beginning of the journey of living a more sober life…they were very helpful at the beginning to kind of get on track. And then when I felt like I could sort of manage it myself, I kind of weaned off of them a little bit.”

Most participants found apps through Google searches, social media ads, or the app store. Only a handful learned about apps in a healthcare setting or from a healthcare professional (eg, in a rehab program and in primary care). Most participants regarded apps as independent of, and not integrated into, other services they might have been receiving. A participant from Delaware said, “I don’t talk to my clinicians about the app. I might talk to them about the things I talk about on the app, but I don’t use the app as part of my counseling that I receive in-person.”

Some participants were not engaged in healthcare or mutual support groups but felt that apps could replace these other forms of support. A participant from Kentucky explained, “Personally, my schedule is pretty busy for going to in-person counseling. So, I do prefer apps for that reason.” These participants perceived apps to be a low-cost and convenient alternative to treatment or other services for problematic drinking.

Among participants who were receiving healthcare services related to their alcohol use (eg, therapy and AUD medication) and/or peer support (eg, Alcoholics Anonymous), most regarded apps as a tool that was most effective when used in combination with other supports. As a participant from Minnesota explained, “It's kind of like a supplement to everything else. I was just trying to drown myself in help.”

Participants reported dozens of app features that they regularly used or appreciated. Some of the most common included tracking the number of drinks consumed, tracking milestones (eg, days sober), giving or receiving support to/from peers (eg, through discussion boards), goal setting and tracking, inspirational content and quotes, tracking accrued benefits of sobriety (weight lost, sleep gained), identifying triggers or patterns around mood and cravings, and general education or examples of sobriety. Participants valued apps for numerous reasons, including anonymity, ongoing positive reinforcement and validation, flexibility and autonomy (as a result of asynchronous features that could be used when and as needed), opportunities for connection with peers, and enhanced ability to identify or quantify behaviors, experiences, and patterns. Participants also mentioned a number of downsides and limitations of the apps they used. These included lack of customization, cost (for some paid apps), burden (eg, entering data and checking multiple apps), and lack of accountability. A participant from Kansas said, “I think a downside is that you can lie to it. You can tell it [the app], ‘I’ve only had 4 drinks today.’ [When in reality, it's like], ‘no you haven’t, you’ve had eight.” Table 3 includes illustrative quotes on benefits and limitations of apps.

**Table 3.** Value and limitations of alcohol reduction apps and illustrative quotes.

Topic	Example quote
Value/benefit	
Anonymity	“It's really good for people who don’t want to put themselves out there… In the beginning, I was like, I don’t want anybody to know that I’m struggling with this. Not that there's any shame in it, I just didn’t want to do it. But that's why the apps are good.”
Ongoing positive reinforcement and validation	“It [specific app] had some more daily motivation features, which I liked. There were different motivational quotes or messages of encouragement that would come up in the app. So, it would kind of give you that fresh outlook every day.”
Flexibility and autonomy	“It made me feel more comfortable than going in-person. It felt like kind of a safe space where I could sort of control the environment.”
Opportunities for connection with peers	“I can talk to other people. That's my most favorite part. So, it's like a sober social network.”
Ability to identify behaviors, experiences, and patterns	“It would ask you how your day was, and I would monitor – I found different patterns within my mood and my thoughts that allowed me to get myself out of ruts, negativity, much quicker. I started to monitor, what was I doing the first day I said it was a good day for me after I said in my app I had a three bad days in a row. It had to do with me exercising. So, I was able to figure out that exercise is the thing that made me sweat. Going on long runs… or going to the gym and lifting weights, that helped me out a lot, actually.”
Downsides/limitations	
Lack of customization	“The [specific app] features don’t change. They don’t seem to evolve in any way throughout your journey, either. So, it's like you’re on this journey and they [apps] stay constant in what they offer and how they are. So that's why I’ve switched between apps.”
Cost	“At first it [specific app] was on a free trial and then you had to pay for it. And that was the main reason why I got rid of it. If it were free or covered by insurance, you would continue to use it? Oh definitely, yes.”
Burden	“It can be a little bit overwhelming, like to check both apps each day can be a lot. That's why I’m doing it every 2 days or so, because it can be a little bit overwhelming to do it every day, just with all of the other features going on the phone. Like, text messages, emails, phone calls.”
Lack of accountability	“[Specific app] is one of the most popular ones, but… to me, it's like, you can lie on those. It's like, ‘I only drink this much.’…I was truthful back when I was drinking, but the accountability wasn’t there.”

This table provides exemplary quotes from interview data organized by the type of value/benefit or limitation of alcohol reduction apps that it illustrates. Source: Authors’ analysis of interview data, October 2024.

The majority of participants did not pay anything out-of-pocket for the apps they used and were not willing to pay for apps. While most of the apps they tried were free or had free trials, a handful of participants paid for apps for some discrete period or used apps that were covered by their employer or insurance provider. Participants mentioned several different reasons they were reluctant to pay out-of-pocket including: (1) being on a fixed income/low income; (2) getting sufficient value from the free features and/or free apps that were available so paying was not needed; and (3) feeling that an individual should only have to pay to interact with a clinician. As 1 participant from Illinois described, “Generally the apps that are related to recovery are free. I would be hesitant to even look at apps with costs because I think there shouldn’t be anything involved with cost in recovery. I don’t think you should be paying for your recovery unless you’re seeing a therapist.”

### Survey findings

**Figure 1.**
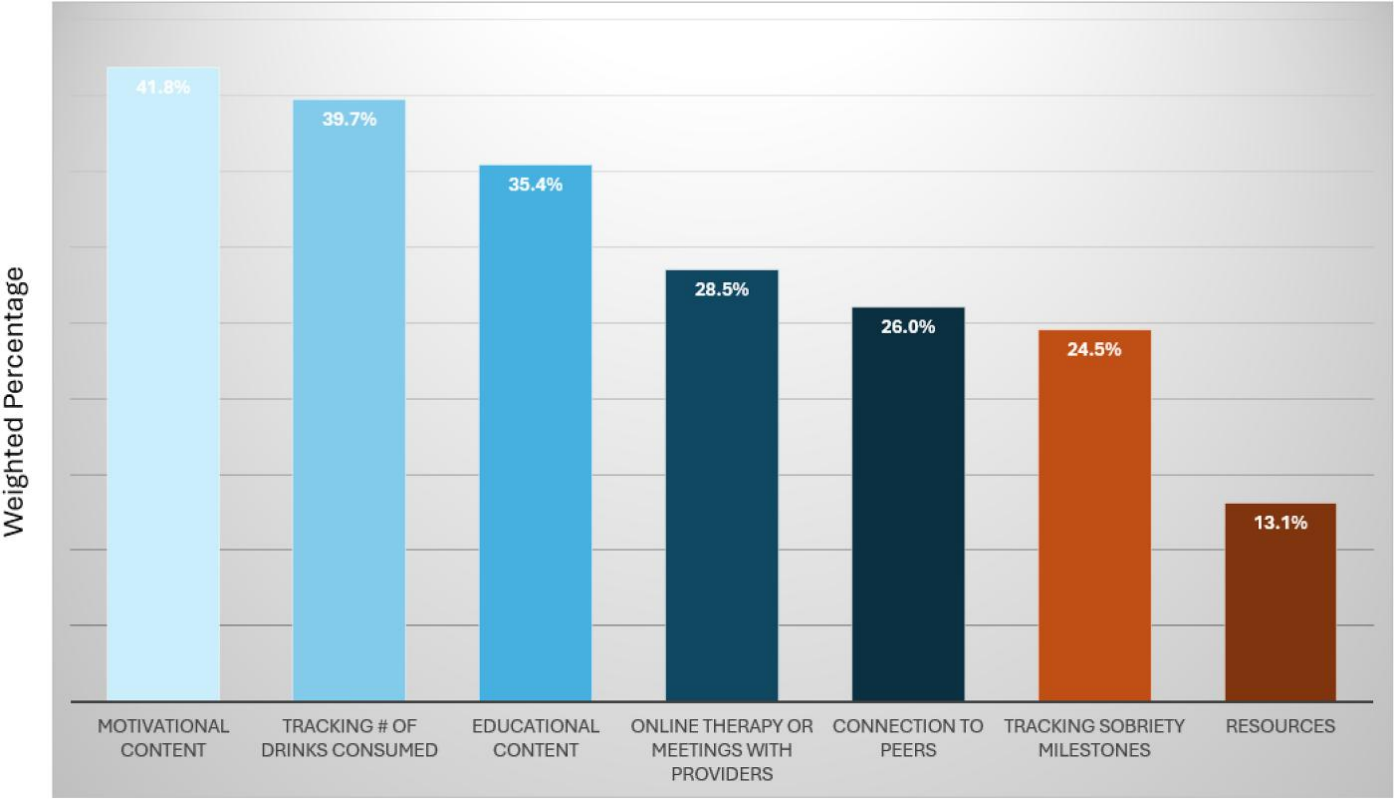
App features that users reported having accessed, 2024. Source: Authors’ analysis of RAND American Life Panel survey data, October 2024. Survey participants were asked which app features they used in the prior 2 years. The sample consisted of 66 individuals. Percentages are weighted.

A total of 66 survey respondents (5.9%, weighted) reported using at least 1 alcohol reduction app in the past 2 years. This suggests that approximately 15 million US adults are using apps for this purpose. Among app users, 55.9% had only used 1 app, and 44.1% had used 2 or more apps (data not shown). The most common features they reported using included: motivational content (41.9%), tracking of drinks consumed (39.7%), and educational content (35.4%) (Figure 1). A total of 63.7% of app users reported that apps were the only tool or support they had used to help with drinking or support sobriety.

## Discussion

We find that approximately 6% of US adults in this nationally representative sample used alcohol reduction apps, and for most, it was the only treatment or support to address alcohol use. While survey respondents represent a heterogenous group with different patterns of alcohol use, it is noteworthy that in national surveys 6% of US adults report heavy drinking in the prior month.^2^ In interviews, some users viewed apps as an alternative to more traditional services and a way to address alcohol use on their own; however, apps were seen as separate even by those who also used treatment or other supports, with minimal disclosure or discussion of app use with their clinicians or in mutual support meetings. App users accessed a variety of app features, with the most common being motivational content, tracking of drinks consumed, and educational content.

Although multiple studies have explored user experiences with specific alcohol reduction apps to inform app design and quality improvement efforts,^17^ this is the first study of which we are aware to describe experiences with alcohol reduction apps more broadly and to calculate prevalence of use. Key benefits of apps such as anonymity, flexibility, and control over how and when to obtain support have been identified in other studies.^10,18^ This study is unique in demonstrating that while app use is common, it is not well-integrated into traditional care or support systems, and apps are largely serving a population that is not engaged in care, even if some users might benefit from additional interventions or tools. Interestingly, although other studies have documented privacy concerns (eg, sharing of data with third parties) with behavioral health apps,^19,20^ this was not a theme in our qualitative data. This is likely the case because we only spoke to active app users.

Results suggest that there are opportunities not only to introduce apps to individuals who are receiving healthcare services and/or participating in mutual support groups, but to reference and embed them in those settings. At minimum, clinicians should expect that many of their patients are experimenting with apps and look for opportunities to leverage those apps to further engage individuals in evidence-based services (eg, therapy and medication). Limited evidence of effectiveness of many publicly-available apps and uncertainty about which apps to recommend are barriers to better integration of apps into healthcare.^9^ Few behavioral health apps are regulated by the Food and Drug Administration as digital therapeutics and available on a prescription basis.^21^ Rather, the vast majority of apps do not provide treatment suggestions and are not considered medical devices subject to regulation. Given widespread interest in apps, it will be important for more research on their relative effectiveness and potentially to develop regulatory pathways and reimbursement to facilitate use of the most promising apps and discourage adoption of ineffective or low value ones. Even smaller-scale changes could facilitate greater integration: some experts have noted that adding a label akin to a nutrition label (ie, which scores popular apps on a variety of criteria) could encourage adoption by clinicians and individuals.^22^

At this point, health apps, including those focused on alcohol reduction, share many similarities with dietary supplements. For many years, the majority of Americans reported taking dietary supplements, but use generally occurred without the awareness of their clinicians. A variety of recommendations now suggest that physicians engage patients by asking about supplement use, discussing available safety and efficacy data, and monitoring for adverse events.^23^ If individuals do more to disclose app use to their clinicians and if clinicians consistently educate themselves and inquire about use, there will be greater opportunities to leverage the strengths of alcohol reduction apps to improve public health.

## Supplementary Material

qxaf034_Supplementary_Data
